# Type 2 Diabetes Mellitus in Patients with Different Types of Thyroid Nodular Lesions Among Western Romanian Patients: A Comprehensive Clinical, Biochemical, and Hormonal Analysis

**DOI:** 10.3390/medicina61071270

**Published:** 2025-07-14

**Authors:** Mervat Matei, Sergiu-Ciprian Matei, Flavia-Medana Petrașcu, Ioana Golu, Melania Balaş, Daniela Amzăr, Ana-Maria Ungureanu, Bianca Roxana Natarâş, Mihaela Maria Vlad

**Affiliations:** 1 Department of Doctoral Studies, “Victor Babeș” University of Medicine and Pharmacy, 300041 Timisoara, Romania; mervat.hassan@umft.ro (M.M.); flavia.petrascu@umft.ro (F.-M.P.);; 2 Department of Endocrinology, Emergency County Hospital Timișoara, 300723 Timișoara, Romania; balas.melania@umft.ro (M.B.); amzar.daniela@umft.ro (D.A.); vlad.mihaela@umft.ro (M.M.V.); 3 Abdominal Surgery and Phlebology Research Centre, “Victor Babeș” University of Medicine and Pharmacy, 300041 Timisoara, Romania; matei.sergiu@umft.ro; 4 Department of Biochemistry, “Victor Babeș” University of Medicine and Pharmacy, 300041 Timisoara, Romania; 5 Department of Internal Medicine II, Discipline of Endocrinology, “Victor Babeș” University of Medicine and Pharmacy, 300041 Timisoara, Romania; 6 Molecular Research Centre in Nephrology and Vascular Pathology, “Victor Babeș” University of Medicine and Pharmacy, 300041 Timisoara, Romania; 7 Department of Radiology and Medical Imaging, “Victor Babeș” University of Medicine and Pharmacy, 300041 Timisoara, Romania; ungureanu.ana-maria@umft.ro; 8 Anapatmol Research Centre, “Victor Babeș” University of Medicine and Pharmacy, 300041 Timisoara, Romania

**Keywords:** thyroid nodules, type 2 diabetes mellitus, free thyroxine, systemic inflammation

## Abstract

*Background and Objectives*: The prevalence of thyroid nodules and type 2 diabetes mellitus (T2D) has increased. This study firstly aims to assess the prevalence of T2DM among thyroid nodules patients who underwent total thyroidectomy in the Western Romanian population. By focusing on the biochemical and hormonal parameters, it also aims to provide insights into possible connections between T2D and different types (benignant or malignant) of thyroid nodules. *Materials and Methods*: A total of 926 patients who underwent total thyroidectomy were analysed, comprising 99 (10.7%) T2D patients and 827 (89.3%) non-diabetic patients (non-T2D). *Results*: This study’s results revealed an increased prevalence of T2D among thyroid nodules patients. Among these patients, higher values of FT4 and inflammatory markers and a higher prevalence of malignant nodules (55%, *p* = 0.001) were noted. *Conclusions*: Our study revealed an increased prevalence of T2D among thyroid nodules patients. The biochemical profile of thyroid nodules in T2D patients presents aspects, like elevated FT4 and inflammatory markers, which should be considered in their clinical management. Furthermore, a T2D patient seems to be more likely to develop thyroid malignancies. Thyroid screening strategies in diabetic patients should be considered.

## 1. Introduction

Thyroid nodules are lesions within the thyroid gland that exhibit structural and functional characteristics that are distinct from surrounding tissues. They are, nowadays, commonly detected in clinical practice using high-resolution ultrasonography [1]. While many thyroid nodules are benign and asymptomatic, their prevalence has been noted to be significantly higher among individuals with metabolic conditions, particularly type 2 diabetes mellitus (T2D) [2,3].

Due to their increasing prevalence and potential for malignancy, the diagnosis and prognosis of thyroid nodules remain a high area of research. Benign subtypes are generally associated with an excellent prognosis. However, malignant nodules, particularly papillary thyroid cancer (PTC), follicular thyroid cancer (FTC), medullary thyroid cancer (MTC), and anaplastic thyroid cancer (ATC), present a higher risk of local invasion, metastasis, and disease recurrence [4]. Among diabetic patients, malignant lesions are more prevalent compared to non-diabetic populations. For instance, PTC, which represents the majority of thyroid cancers, is frequently identified in T2D patients and is often associated with more aggressive clinical features, such as larger tumour size and higher rates of lymph node involvement [5,6].

The formation of thyroid nodules is affected by a complex interaction of genetic factors, metabolic irregularities, and environmental influences, with each element playing a distinct role in their development and advancement. For individuals with T2D, thyroid nodules are notably more prevalent, with research reporting a 60% prevalence among T2D patients compared to 43% in individuals without diabetes [7].

Thyroid dysfunction and T2D are among the most common chronic endocrine disorders globally, with notable overlaps in prevalence and clinical implications [8]. Research has increasingly highlighted the bidirectional relationship between these conditions, emphasising how each can influence the onset, progression, and outcomes of the other. For example, thyroid dysfunction is more prevalent in patients with diabetes than in the general population, with T2D frequently coexisting with hypothyroidism [8,9].

The mechanisms linking these conditions involve complex interactions between metabolic, hormonal, and immune pathways. In patients with T2D, insulin resistance and chronic hyperglycaemia can influence thyroid hormone metabolism and lead to thyroid dysfunction [9,10].

Oxidative stress (OS) is a defining feature of T2D and is based on an imbalance between reactive oxygen species (ROS) production and the body’s antioxidant defence systems. In T2D, chronic hyperglycaemia triggers ROS overproduction, promoting cellular and tissue damage [11,12]. Due to its high oxidative metabolic activity and dependence on ROS for hormone synthesis, the thyroid gland is particularly vulnerable to oxidative damage [13].

During hyperglycaemia, the mitochondrial respiratory chain becomes a significant source of ROS. ROS result in the oxidative damage of lipids, proteins, and nucleic acids, which, in turn, activate inflammatory signalling pathways. This inflammation further worsens insulin resistance, creating a self-reinforcing cycle of OS and metabolic disruption [14].

In addition, the thyroid hormone synthesis process generates ROS as a by-product, making it rely on a robust antioxidant defence to prevent self-inflicted oxidative damage. In conditions of increased systemic inflammation, the antioxidant capacity of the thyroid is overwhelmed, contributing to dysfunction and nodule formation [13,14,15].

Despite extensive research on the individual roles of T2D, systemic inflammation, and hormonal dysregulation in thyroid health, the combined impact of these factors remains to be fully understood. Current findings indicate that the interaction between metabolic processes, hormonal activities, and inflammatory responses could have an impact on the formation and behaviour of thyroid nodules among diabetic individuals. It is crucial to comprehend these connections to enhance the precision of diagnosis, manage risks effectively, and treat thyroid nodules appropriately [16,17]. No such studies had been conducted in Eastern Europe.

This study intends to consolidate the current knowledge by firstly examining the prevalence of T2D among thyroid nodules patients in the Western Romanian population and subsequently analysing the characteristics of thyroid nodules in patients with and without T2D.

## 2. Materials and Methods

Study design, patients, and data collection. The present paper represents retrospective descriptive and analytical study, and was conducted in “Pius Brînzeu” Emergency County Hospital Timișoara, the largest county hospital in Western Romania, which serves patients from the entire region. The medical records of 1097 patients followed up in the Endocrinology Department, “Pius Brînzeu” Emergency County Hospital Timișoara, Romania, who were diagnosed with thyroid nodules and subsequently admitted to this hospital and operated on in the 1st Surgical Department between January 2017 and January 2024, were evaluated (all the patients diagnosed with thyroid nodules during the defined period were initially analysed). Patients’ clinical charts, paraclinical investigations, surgical records, and pathological results were researched. The following data were collected in order to be statistically analysed: demographic data including age, gender, and native environment (urban/rural); clinical data including body mass index (BMI) and diabetes mellitus diagnosis; laboratory tests including complete blood count (CBC) and inflammatory markers: red blood cell count (RBC), white blood cell count (WBC) with WBC differential (neutrophils, lymphocytes, eosinophils, basophils, and monocytes), platelet count (PLT), erythrocyte sedimentation rate (ESR), and fibrinogen; hormonal profile and additional biochemical parameters including free triiodothyronine (FT3), free thyroxine (FT4), thyroid stimulating hormone (TSH), total protein (TP), blood sugar (glycaemia), glycated haemoglobin (HbA1C), and creatinine; surgery type (thyroid lobectomy, subtotal or total thyroidectomy); and pathological result. Blood samples were collected in the morning (between 7:00 and 9:00 a.m.) after an overnight fast to minimise diurnal variations and ensure the accurate measurement of biochemical and hormonal parameters. Standardised reference ranges were used in accordance with the hospital laboratory guidelines.

Considering the studied paraclinical parameters, the laboratory reference values were as follows: RBC, 4.5–5.9 × 10^6^/µL; WBC, 4–9.5 × 10^3^/µL; neutrophils, 1.8–6.7/*10^3^/µL; lymphocytes, 0.8–3.8/*10^3^/µL; eosinophils, 0–0.4/*10^3^/µL; basophils, 0–0.1/*10^3^/µL; monocytes, 0.1–0.9/*10^3^/µL; PLT, 150–400 × 10^3^/µL; ESR, 0–15 mm/h; fibrinogen, 200–393 mg/dL; FT3, 3.54–6.47/pmol/L; FT4, 11.50–22.70/pmol/L; TSH, 0.55–4.78/mIU/L; TP, 6.4–8.2/g/dL; glycaemia, 74–106 mg/dL; HbA1C, 5.7–6.4%; and creatinine, 0.7–1.2/mg/dL.

Obesity was defined and categorised according to the World Health Organization (WHO) guidelines. Body mass index (BMI), calculated as weight in kilograms divided by the square of height in meters (kg/m^2^), was used to classify weight status: normal weight: 18.5–24.9 kg/m^2^; overweight: 25.0–29.9 kg/m^2^; obesity Class I: 30.0–34.9 kg/m^2^; obesity Class II: 35.0–39.9 kg/m^2^; obesity Class III: ≥40.0 kg/m^2^ [18].

Enrolment criteria. All the patients admitted for surgery during the set time period were initially analysed. The indications for surgery were established by a team made up of endocrinologists and surgeons, and the type of the surgery performed was chosen according to existing indications from guidelines and standard protocols. The reasons for total thyroidectomy followed the standard protocol considerations as follows: large thyroid nodules; thyroid nodules with ultrasound characteristics of malignancy, or which change their characteristics during follow-up; compressive thyroid nodules or goitre; thyroid goitre with poor conservative management; fine-needle aspiration biopsy with malignant result; and intraoperative surgical considerations (suspicion of lesion extension to the contralateral lobe, bleeding, etc.). All patients had a normal thyroid checkup before surgery.

In order to maintain the uniformity in the study groups, only patients in which total thyroidectomy was performed were included in this analysis.

To ensure uniformity in hormonal profile analysis, patients who were receiving thyroid hormone replacement therapy (levothyroxine) or antithyroid medication (methimazole, propylthiouracil) were excluded from the study. This exclusion criterion was applied to prevent potential confounding effects on thyroid function parameters. Additionally, all patients included in the study were identified at the early stages of their disease before initiating any thyroid-related medication. A small number of patients who had already started treatment were excluded to maintain the homogeneity of the cohort.

**Figure 1:**
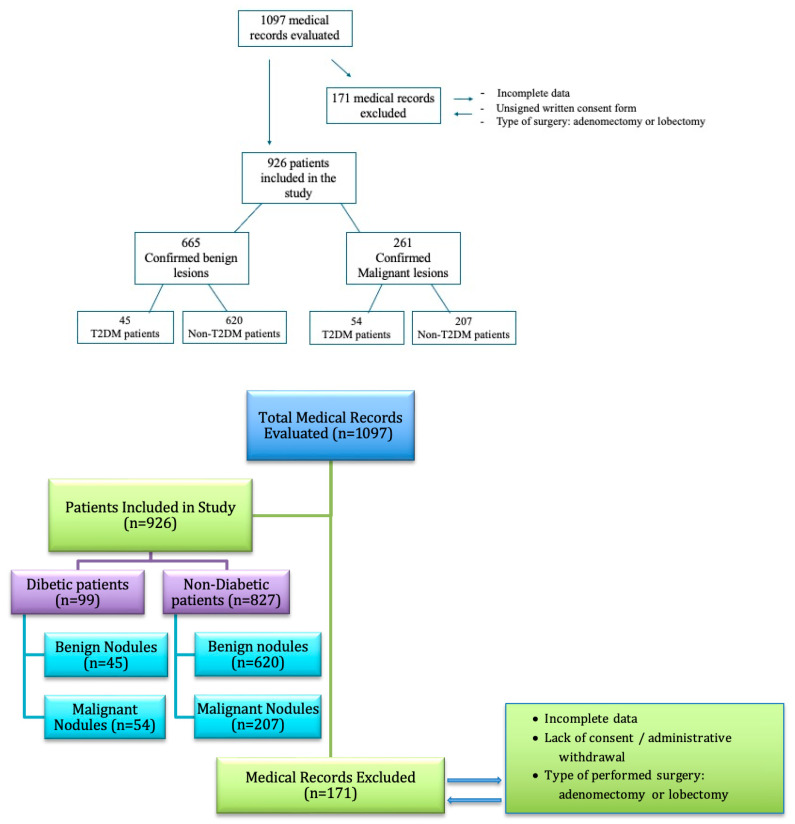
Chart diagram of patient enrolment.

Also, the following cases were excluded from the study: patients with incomplete data and patients who did not sign the written informed consent form in order to participate in the study. Subsequently, by applying these criteria, 926 patients were analysed. Of these, there were 99 T2D patients and 827 non-diabetic (non-T2D) patients. The chart diagram of patient enrolment is presented in Figure 1.

Statistical analysis. Statistical analyses were performed using MedCalc^®^ Statistical Software version 20.118 (MedCalc Software Ltd., Ostend, Belgium; 2022). Continuous variables were assessed for normality using the Kolmogorov–Smirnov test, and those with a normal distribution were expressed as means ± standard deviations. Categorical variables were expressed as frequencies and percentages. Comparisons between patients with and without type 2 diabetes mellitus (T2D and non-T2D) were conducted using the independent sample t-test for continuous variables (e.g., age) and the chi-square test for categorical variables (e.g., gender and locality). A *p*-value of <0.05 was considered statistically significant. The results were further evaluated at a 95% confidence interval. Adjusted *p*-values were calculated accordingly. This comprehensive approach ensured the robust evaluation and interpretation of the data.

## 3. Results

A total of 926 patients who underwent total thyroidectomy were included in the study, comprising 99 (10.7%) T2D patients and 827 (89.3%) non-T2D patients. The analysis focused on comparing demographic, clinical, and biochemical parameters between these two groups, as shown in Table 1.

Among T2D patients, the majority were female (91.92%), with male patients accounting for only 8.08%. Similarly, in the non-T2D group, females comprised 91.02% and males 8.98%. Regarding living environment, 62.63% of T2D patients resided in urban areas, while 37.37% were from rural settings. In contrast, 67.48% of non-T2D patients were urban residents and 32.52% were rural residents, with a marginally significant difference in distribution (*p* = 0.047). The mean age of T2D patients was significantly higher at 62.01 ± 9.04 years compared to 54.31 ± 12.31 years in the non-T2D group (*p* < 0.01). BMI was also significantly elevated among T2D patients (31 ± 5 kg/m^2^) compared to their non-diabetic counterparts (25 ± 4 kg/m^2^, *p* < 0.01). Obesity was far more prevalent among T2D patients, with higher proportions observed across all obesity classes. Normal-weight individuals represented a smaller fraction in the T2D group, while severe obesity (Classes II and III) was significantly more frequent compared to non-T2D patients.

Biochemically, T2D patients exhibited significantly higher glycemia levels (140 ± 30 mg/dL) compared to non-T2D patients (95 ± 10 mg/dL, *p* < 0.01). HbA1c levels were similarly elevated in T2D patients (8.5 ± 1.2%) versus non-T2D patients (5.5 ± 0.5%, *p* < 0.01). Moreover, creatinine levels were slightly higher in T2D patients (1.2 ± 0.3 mg/dL) compared to non-T2D patients (1.0 ± 0.2 mg/dL, *p* < 0.05), potentially reflecting early renal involvement.

The analysis of haematological and inflammatory parameters (Table 2) revealed significant differences between T2D and non-T2D patients. WBC counts were significantly elevated in T2D patients (8.53 ± 1.98 × 10^3^/µL) compared to non-T2D patients (7.57 ± 1.88 × 10^3^/µL, *p* = 9.869). Similarly, neutrophil levels were higher in T2D patients (5.56 ± 1.65 × 10^3^/µL) than in non-T2D patients (4.78 ± 1.61 × 10^3^/µL, *p* = 1.655). Other parameters, such as RBC counts (4.65 ± 0.43 × 10^6^/µL in T2D vs. 4.64 ± 0.40 × 10^6^/µL, *p* = 0.862), lymphocyte counts (2.25 ± 0.74 × 10^3^/µL in T2D vs. 2.14 ± 0.73 × 10^3^/µL in non-T2D, *p* = 0.146), PLT (281.22 ± 69.37 × 10^3^/µL in T2D vs. 269.36 ± 65.72 × 10^3^/µL in non-T2D, *p* = 0.108), and TP (7.1 ± 1.3 g/dl in T2D vs. 7.4± 0.96 g/dL in non-T2D, *p* = 0.059), did not show statistically significant differences between the groups. Markers of systemic inflammation showed notable differences. ESR was significantly elevated in T2D patients (28.94 ± 10.89 mm/h) compared to non-T2D patients (20.96 ± 6.71 mm/h, *p* = 4.528). Fibrinogen levels were also significantly higher in T2D patients (377.61 ± 87.99 mg/dL) compared to non-T2D patients (354.78 ± 88.15 mg/dL, *p* = 0.016).

The analysis of thyroid hormonal parameters (Table 1) revealed significant differences in TSH and FT4 levels between T2D and non-T2D patients, while no statistically significant differences were observed for FT3 levels. Patients with T2D exhibited significantly lower TSH levels (1.62 ± 1.24 mIU/L) compared to non-T2D patients (1.96 ± 2.69 mIU/L, *p* = 0.032). This suggests a potential alteration in thyroid function regulation associated with T2D. FT4 levels were significantly elevated in the T2D group (16.06 ± 2.72 pmol/L) compared to the non-T2D group (14.79 ± 3.87 pmol/L, *p* < 0.001). In contrast, FT3 levels did not differ significantly between the two groups (4.93 ± 0.93 pmol/L in T2D vs. 5.05 ± 0.90 pmol/L in non-T2D, *p* = 0.239).

The histopathological findings are summarised in Table 3, highlighting distinct differences between T2D and non-T2D patients. Malignant nodules were more prevalent in the T2D group (55%) compared to the non-T2D group (25%), with an odds ratio of 3.59 (95% CI: 2.35–5.50; *p* = 0.001). Conversely, benign nodules were more frequent in the non-T2D group (75%) than in the T2D group (45%), with an odds ratio of 0.86 (95% CI: 0.69–1.03; *p* = 0.03).

**Figure 2:**
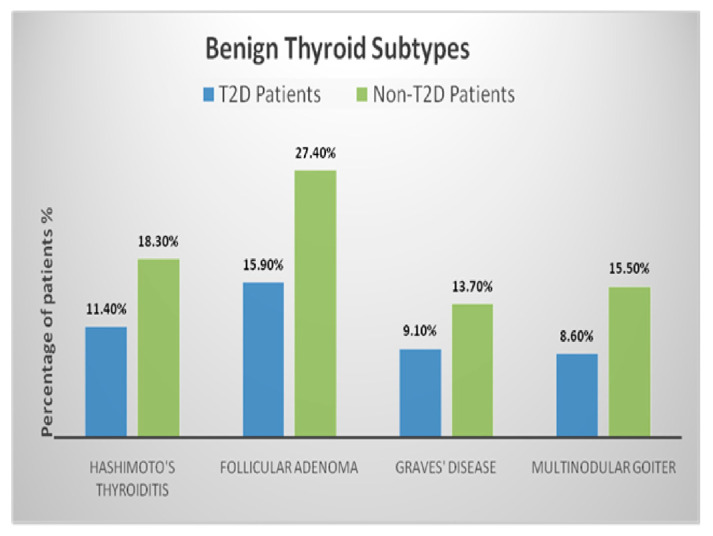
Distribution of benign thyroid subtypes among T2D and non-T2D patients. The chart displays the proportion of patients with each histopathological subtype within the benign categories.

**Figure 3:**
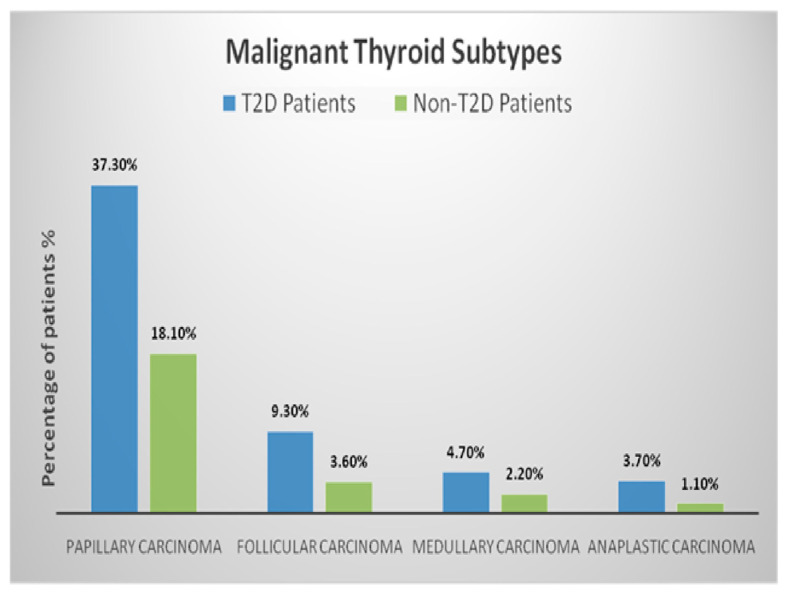
Distribution of malignant thyroid subtypes among T2D and non-T2D patients. The chart displays the proportion of patients with each histopathological subtype within the malignant categories.

The distribution of benign and malignant thyroid subtypes in T2D and non-T2D patients, as illustrated in Figure 2 and Figure 3, reveals notable differences in histopathological patterns. Among benign subtypes, follicular adenomas accounted for 15.9% of T2D patients compared to 27.4% of non-T2D patients. Hashimoto’s thyroiditis was present in 11.4% of T2D patients versus 18.3% of non-T2D patients. Graves’ disease and multinodular goitre showed distributions of 9.1% and 8.6% in T2D patients, respectively, while these subtypes were observed at 13.7% and 15.5% in non-T2D patients. Overall, benign nodules constituted 45% of nodules in T2D patients and 75% in non-T2D patients, with a significantly reduced likelihood of benign nodules in the T2D group (adjusted OR: 0.86, 95% CI: 0.69–1.03; *p* = 0.03). For malignant subtypes, PTC was the most frequent, representing 37.3% of T2D patients compared to 18.1% of non-T2D patients. FTC was present in 9.3% of T2D patients and 3.6% of non-T2D patients. MTC and ATC were observed at rates of 4.7% and 3.7% in T2D patients compared to 2.2% and 1.1% in non-T2D patients, respectively. Malignant nodules were significantly more prevalent in T2D patients, accounting for 55% of nodules compared to 25% in non-T2D patients, with an adjusted odds ratio of 1.17 (95% CI: 0.94–1.41; *p* = 0.01).

A statistical analysis using multivariate logistic regression demonstrated that T2D patients had a higher likelihood of developing malignant nodules, particularly PTC (adjusted OR: 1.25, 95% CI: 1.05–1.48; *p* = 0.01). In contrast, benign subtypes, such as follicular adenomas, were significantly less frequent in the T2D group, further emphasising the impact of diabetes on thyroid pathology. These findings underscore the necessity of rigorous evaluation and tailored management strategies for thyroid nodules, especially in diabetic populations.

To account for multiple comparisons, we applied the Holm–Bonferroni correction to adjust the significance threshold. We decided to include in the table only the variables with an original *p*-value ≤ 0.05, as these were initially considered statistically significant. After adjustment, FT4 (adjusted *p* = 0.01), malignant nodules (adjusted *p* = 0.01), age (adjusted *p* = 0.05), BMI (adjusted *p* = 0.05), HbA1c (adjusted *p* = 0.05), and glycemia (adjusted *p* = 0.05) remained statistically significant. In contrast, other variables, including TSH (adjusted *p* = 0.16), fibrinogen (adjusted *p* = 0.08), benign nodules (adjusted *p* = 0.15), and creatinine (adjusted *p* = 0.25), did not retain statistical significance after adjustment. The detailed results are presented in Table 4.

## 4. Discussion

The results revealed that the presence of T2D is associated with an increased risk of malignant thyroid nodules. Data from the literature indicate a prevalence of thyroid diseases in diabetic patients that is 2–3 times higher than in non-diabetic subjects; it increases with age and is strongly influenced by female gender and autoimmune disease [8,9]. However, no type 1 diabetes (T1D) patient was noted in our cohort. T1D is more often linked to autoimmune thyroid disease, with this association being frequently noted in the younger population [19,20] and even in children [21].

In this analysis, all the patients who met the inclusion criteria during the set time period were included, resulting a significant difference in the ages of the two study groups. The main concern regarding this result might be related to considerations about age as a risk factor, especially in developing malignancies. However, while the risk of developing malignant lesions increases with age, more solid data supporting the association between T2D and cancers are available [22], causing us to not consider age as a confounding factor but to support the existing literature statements regarding the relationship between diabetes mellitus and cancers [23].

Significant differences in the hormonal profile between T2D and non-T2D patients were noted in this analysis. The pleiotropic effects of thyroid hormones on various metabolic processes are well known. Uncontrolled hyperthyroidism in diabetic patients may trigger hyperglycaemic emergencies, while recurrent hypoglycaemic episodes have been reported in diabetic patients with hypothyroidism. Furthermore, thyroid dysfunction may amplify cardiovascular disease risk in diabetic patients through inter-relationships with dyslipidaemia, insulin resistance, and vascular endothelial dysfunction [24]. In addition, the metabolic and hormonal impairment in T2D patients can further complicate their clinical management. Hyperglycaemia and insulin resistance may delay wound healing and increase the risk of post-operative complications in patients who underwent thyroidectomy [25], while coexisting metabolic conditions such as obesity exacerbate the disease burden.

Moreover, thyroid dysfunction, as evidenced by altered levels of TSH and FT4, is more common in diabetic patients and may further contribute to adverse outcomes [26,27].

The hypothalamic–pituitary–thyroid (HPT) axis plays an important role in metabolism and energy balance, and dysregulation within this axis can influence insulin sensitivity and glucose homeostasis. Thyroid hormones have insulin-antagonistic effects in the liver, whereas they act synergically with insulin in the peripheral tissues [28]. Insulin resistance may occur in both hyperthyroidism and hypothyroidism status [29]. In hyperthyroidism, thyroid hormones increase the endogenous glucose production and insulin requirement, alongside decreasing hepatic insulin sensitivity, which may lead to hepatic insulin resistance [28]. On the other hand, in hypothyroidism, the insulin resistance of peripheral tissues prevails. Furthermore, these effects seem not to be restricted only to overt hypothyroidism or hyperthyroidism, but might also appear in subclinical disorders, or even alterations of hormone levels in the reference range [30].

Insulin resistance has been widely recognised as a potential contributor to thyroid malignancy. High insulin levels found in prediabetes and early T2D can stimulate thyroid tissue hyperplasia, leading to thyroid enlargement and nodule formation [31]. It can promote cell proliferation and inhibit apoptosis through the insulin and insulin-like growth factor (IGF) pathways, which may facilitate tumorigenesis in the thyroid gland. Multiple studies have reported an increased prevalence of insulin resistance among patients with thyroid cancer, especially those with papillary thyroid carcinoma (PTC) [32]. A recent meta-analysis highlighted the link between insulin resistance and thyroid carcinoma, revealing that both elevated fasting serum insulin levels and insulin resistance are associated with a higher risk of developing thyroid cancer [33].

Elevated glycaemia, HbA1C, and inflammatory markers (WBC, neutrophils, ESR, and fibrinogen) in T2D patients highlight the role of chronic systemic inflammation in modulating thyroid disease outcomes. Diabetes, particularly T2D, is characterised by chronic low-grade inflammation, which can activate inflammatory pathways like MAPK and can lead to the increased production of inflammatory cytokines such as TNF-a, IL-6 and IL-8 that promote thyroid cell proliferation, inhibit apoptosis, and enhance angiogenesis, all of which are conductive to tumour growth.

Furthermore, systemic inflammation is often observed in patients with T2D, which contributes to insulin resistance and impacts the thyroid gland [29]. Systemic inflammation is a contributing factor to the increased prevalence of thyroid malignancy, but it does not, however, fully explain it on its own [34]. Thyroid malignancy is a multifactorial disease, which includes possible genetic mutations, chromosome alterations, environmental factors like radiation exposure (especially during childhood), environmental pollutants, and chronic iodine deficiency, without disregarding the modern improved detection methods with enhanced and wider available ultrasound screening and more frequent fine-needle aspiration biopsies [35,36].

The results of this analysis revealed significant differences between groups in terms of inflammatory markers. Increased pro-inflammatory status is common to malignant lesions, but it is also a risk factor for their occurrence [37].

Insulin resistance and compensatory hyperinsulinemia stimulate proliferative pathways via the IGF-1 and insulin receptors [31], promoting cellular growth and inhibiting apoptosis in thyroid follicular cells [38]. The recent literature suggests a possible link between sustained IGF-1 stimulation and the pathogenesis of papillary thyroid carcinoma, the most prevalent subtype in our T2D cohort [39].

OS plays a central role in the pathophysiology of both T2D and thyroid malignancy. OS is a key factor in the onset and progression of diabetes, especially T2D. Persistent hyperglycaemia results in the excessive generation of reactive oxygen species (ROS), which surpass the body’s antioxidant defence capacity. This imbalance causes damage to cellular structures and DNA, playing a significant role in the development of insulin resistance, beta-cell dysfunction, and vascular complications [40]. Similarly, in the thyroid gland, elevated OS has been shown to be implicated in DNA damage, genomic instability, and the progression of thyroid cancer, particularly PTC [41].

The interplay between diabetes and thyroid malignancy may be mediated through shared oxidative pathways. The NADPH oxidase (NOX) enzymes, known for producing reactive oxygen species (ROS), have been associated with both diabetes and thyroid cancer [42]. In thyroid malignancies, their activity contributes to tumour cell growth and invasion. This overlap in oxidative mechanisms suggests that OS may serve as a common underlying factor in the development of both conditions [43].

Although most thyroid nodules in T2D patients are benign, the interplay of oxidative stress, chronic inflammation, and hormonal imbalance increases the risk of malignancy [15,44,45]. A higher percentage of malignant nodules were encountered in the T2D group (55% compared to 25%), underscoring the influence of diabetes on thyroid pathology. Additionally, a higher frequency of PTC and lower prevalence of benign subtypes, such as follicular adenomas, in T2D patients suggests a distinct pattern of thyroid pathology influenced by diabetes. Further prospective studies are needed to explore this aspect.

Thyroid hormone abnormalities were noted, such as hypothyroidism or hyperthyroidism, which can exacerbate glycaemia control challenges in diabetic patients by altering insulin sensitivity and glucose metabolism [10,11].

Increased TSH and reduced FT3 levels are associated with diabetic kidney disease in T2D patients in a sex-dependent manner [46]. Peripheral deiodinase enzymes, especially type 1 and type 2 (DIO1 and DIO2), play a vital role in converting thyroxine (T4) into its active form, triiodothyronine (T3). It is well documented that inflammatory cytokines and oxidative stress, both commonly elevated in T2D, can hinder the activity of these enzymes. This disruption may result in lower-than-expected T3 levels, even when T4 levels are normal or elevated. Such a mechanism could account for the biochemical pattern observed in our T2D cohort, where FT4 levels were significantly increased while FT3 levels remained relatively stable.

Non-thyroidal illness syndrome (NTIS) is frequently observed in patients with systemic illness, including T2D, and is thought to represent an adaptive response to metabolic stress [47]. A large cross-sectional study found that decreased FT3, decreased FT3/FT4 ratios, and increased FT4 levels were independently related to a higher prevalence of T2D in both males and females [48]. Although our patient cohort consisted of preoperative cases rather than critically ill individuals, chronic metabolic and inflammatory stress may nonetheless contribute to alterations in thyroid hormone levels consistent with NTIS.

Additionally, thyroid hormone levels, especially FT4 levels, were significantly negatively correlated with diabetic kidney disease in T2D patients [49]. Chronic kidney disease (CKD) is known to disrupt both the metabolism and clearance of thyroid hormones, potentially intensifying biochemical patterns resembling NTIS. Specifically, reduced renal function can impair deiodinase activity, leading to decreased T3 levels and further complicating the thyroid hormone profile in individuals with diabetes.

A recent study revealed that patients with T2D who had normal thyroid function but low FT3/FT4 levels showed a significantly higher incidence of diabetic kidney disease. Furthermore, low FT3 levels and a reduced FT3/FT4 ratio were identified as independent risk factors for diabetic kidney disease [50].

Moreover, iodine intake represents an important influence on the development and progression of thyroid nodular disease. Although direct iodine measurements were not available for the participants in our study, the mandatory use of iodised salt in Romania, following the salt iodisation policies implemented in the early 2000s, covering both households and the bakery sector, appears to provide adequate iodine levels in adults. Based on the most recently published national data, while comparing the distribution of median urinary iodine concentration in eight regions of Romania, in West Romania, the cutoff for iodine sufficiency was met [51]. Also, recent evidence suggests that T2D may influence iodine metabolism and urinary iodine excretion potentially through renal alterations or dietary modifications [52]. These interactions may have implications for thyroid function and nodular pathology in diabetic patients and represent a direction for future prospective research.

Routine annual thyroid testing should be targeted in diabetic patients. Also, considering the bidirectional relationship between T2D and thyroid dysfunction, some implications regarding thyroid nodule management should be addressed. Additionally, for medication and dietary measures, considering the delayed healing process often encountered in T2D patients, increased care should be taken, especially if these patients are candidates for surgery.

Considering the variability of subtypes of thyroid nodular lesions, as well as the possibility of synchronous thyroid tumour occurrence, for better result accuracy, only patients who underwent total thyroidectomy and for whom an entire specimen of pathological examination was provided were included in this study. While ultrasound, contrast-enhanced ultrasonography, elastography, and fine-needle aspiration cytology are useful tools in detecting thyroid nodules that raise malignancy suspicion [53,54,55,56], only the pathological exam establishes a diagnosis of complete certainty, which also plays a role in prognostic assessment. Histopathological examination remains the cornerstone for distinguishing between benign and malignant thyroid lesions, as well as identifying specific subtypes. Incorporating histopathological and immunohistochemical assessments into clinical practice is essential for a nuanced understanding of thyroid tumours [57,58]. These methods not only refine diagnostic accuracy, but also pave the way for personalised therapeutic approaches by identifying molecular markers like Ki-67, E-cadherin [59], or vascular endothelial growth factor (VEGF) and pathways that can be targeted effectively [60,61]. Such integrative strategies hold promises for improving outcomes in patients with complex thyroid malignancies.

Despite the fact that this paper brings valuable information, some limitations should be considered, the main one being the retrospective nature of this study followed by the lack of a wider data panel. Additional data like vitamin D, lipid profile, and albumin [35,62,63,64], which could not be provided for all the patients, would have added substance to this analysis. Additionally, although we excluded patients who were receiving thyroid hormone replacement or antithyroid drugs at the time of blood sampling to reduce confounding, future prospective studies should incorporate medication data to explore these relationships more thoroughly. However, given the topicality of the issue, further prospective and multicentre studies should be considered. As well, thyroid disease screening strategies in routine diabetes care should be considered [65,66].

## 5. Conclusions

By focusing on biochemical and hormonal parameters, this study provides insights into possible connections between T2D and different types (benignant or malignant) of thyroid nodules, serving as a foundation for future investigations. The prevalence of malignant thyroid nodules is increased in patients with T2D compared to non-T2D individuals. The biochemical profile of thyroid nodules in T2D patients presents particular aspects, like elevated FT4 and inflammatory markers, which should be considered in their clinical management. Furthermore, a T2D patient seems to be more likely to develop thyroid malignancies. Thyroid screening strategies in diabetic patients are recommended.

## Figures and Tables

**Table 1:** Demographic and clinical characteristics of patients.

Characteristic	T2D Patients *n* = 99 (Men ± SD/%)	Non-T2D Patients *n* = 827 (Men ± SD/%)	*p*-Value
Age (years)	62.01 ± 9.04	54.31 ± 12.31	<0.01
Gender (F %)	91.91	91.01	0.900
Living Environment (Urban%)	62.62%	67.47	0.47
BMI (kg/m^2^)	31 ± 5	25 ± 4	<0.01
Normal Weight %	10	40	0.01
Overweight %	30	35	
Obesity Class I %	25	15	
Obesity Class II %	20	8	
Obesity Class III %	15	2	
Glycemia (mg/dL)	140 ± 30	95 ± 10	<0.01
HbA1c %	8.5 ± 1.2	5.5 ± 0.5	<0.01
Creatinine (mg/dL)	1.2 ± 0.3	1.0 ± 0.2	0.05
FT3 (pmol/L)	4.93 ± 0.92	5.04 ± 0.900	0.238
FT4 (pmol/L)	16.05 ± 2.71	14.78 ± 3.868	<0.001
TSH (mIU/L)	1.62 ± 1.24	1.95 ± 2.69	0.032

Statistically significant differences between T2D (type 2 diabetes mellitus) and non-T2D (non-type 2 diabetes mellitus) groups (*p* < 0.05). BMI = body mass index (normal range: 18.5–24.9 kg/m^2^), glycemia (normal range: 70–99 mg/dL), HbA1c (normal range: <5.7%), creatinine (normal range: 0.6–1.2 mg/dL), FT3 = free triiodothyronine (normal range: 3.54–6.47/pmol/L), FT4 = thyroxine (normal range: 11.50–22.70/pmol/L), and TSH = thyroid-stimulating hormone (normal range: 0.55–4.78/mIU/L).

**Table 2:** Haematological and inflammatory parameters.

Parameter	T2D Patients *n* = 99 (Men ± SD)	Non-T2D Patients *n* = 827 (Men ± SD)	*p*-Value
RBC (×10^6^/µL)	4.65 ± 0.43	4.64 ± 0.40	0.862
WBC (×10^3^/µL)	8.53 ± 1.98	7.57 ± 1.88	9.869
Neutrophils (×10^3^/µL)	5.56 ± 1.65	4.78 ± 1.61	1.655
Lymphocytes (×10^3^/µL)	2.25 ± 0.74	2.14 ± 0.73	0.146
PLT (×10^3^/µL)	281.22 ± 69.37	269.36 ± 65.72	0.108
ESR (mm/h)	28.94 ± 10.89	20.96 ± 6.71	4.528
Fibrinogen (mg/dL)	377.61 ± 87.99	354.78 ± 88.15	0.016

Statistically significant difference between T2D (type 2 diabetes mellitus) and non-T2D (non-type 2 diabetes mellitus) groups (*p* < 0.05). RBC= red blood cells count (4.5–5.9 × 10^6^/µL), WBC= white blood cells count (4.0–9.5 × 10^3^/µL), neutrophils (1.8–6.7 × 10^3^/µL), lymphocytes (0.8–3.8 × 10^3^/µL), PLT = platelet count (150–400 × 10^3^/µL), ESR = erythrocyte sedimentation rate (0–15 mm/h), and fibrinogen (200–393 mg/dL).

**Table 3:** Histopathological findings.

Characteristic	T2D Patients (%) *n* = 99	Non-T2D Patients (%) *n* = 827	Odds Ratio (95% CI)	*p*-Value
Malignant Nodules	55% (*n* = 54)	25% (*n* = 207)	3.59 (2.35–5.50)	0.001

Statistically significant difference in histopathological patterns between T2D (type 2 diabetes mellitus) and non-T2D (non-type 2 diabetes mellitus) groups (*p* < 0.05).

**Table 4:** Holm–Bonferroni-adjusted *p*-values for multiple comparisons in the study population.

Test	Original *p*-Value	Holm–Bonferroni-Adjusted *p*-Value
Age (years)	<0.01	0.05
BMI (kg/m^2^)	<0.01	0.05
HbA1c %	<0.01	0.05
Glycemia	<0.01	0.05
Creatinine (mg/dL)	0.05	0.25
FT4	<0.001	0.01
TSH	0.032	0.16
Fibrinogen (mg/dL)	0.016	0.08
Benign Nodules	0.03	0.15
Malignant Nodules	0.001	0.01

The original *p*-values and the adjusted *p*-values after applying the Holm–Bonferroni correction for multiple comparisons. Statistically significant associations, adjusted *p* value ≤ 0.05. BMI = body mass index, HbA1c, glycemia, FT4 = thyroxine, TSH = thyroid-stimulating hormone, fibrinogen, benign nodules, malignant nodules.

## Data Availability

The datasets used and/or analysed during the current study may be requested from the corresponding author.

## References

[1] Zhang F., Li Y., Yu X., Wang X., Lin Z., Song B., Tian L., Feng C., Shan Z., Teng W. (2021). The Relationship and Gender Disparity Between Thyroid Nodules and Metabolic Syndrome Components Based on a Recent Nationwide Cross-Sectional Study and Meta-Analysis. Front. Endocrinol..

[2] Cao C., Li C., Li X., Sun W., Wang Y. (2023). Association of systemic immune-inflammation index (SII) and aggregate index of systemic inflammation (AISI) with thyroid nodules in patients with type 2 diabetes mellitus: A retrospective study. BMC Endocr. Disord..

[3] Tamez-Pérez H.-E., Martínez E., Quintanilla-Flores D.L., Tamez-Peña A.L., Gutiérrez-Hermosillo H., Díaz de León-González E. (2012). The rate of primary hypothyroidism in diabetic patients is greater than in the non-diabetic population: An observational study. Med. Clin..

[4] Miao S., Jing M., Sheng R., Cui D., Lu S., Zhang X., Jing S., Zhang X., Shan T., Shan H. (2020). The analysis of differential diagnosis of benign and malignant thyroid nodules based on ultrasound reports. Gland Surg..

[5] Dong W., Zhang L., Huang X., Xu F., Chen G., Zheng W. (2022). Different types of diabetes mellitus and risk of thyroid cancer: A meta-analysis of cohort studies. Front. Endocrinol..

[6] Kitahara C.M., Schneider A.B. (2022). Epidemiology of thyroid cancer. Cancer Epidemiol. Biomark. Prev..

[7] Zhang H.-M., Feng Q.-W., Niu Y.-X., Su Q., Wang X. (2019). Thyroid nodules in type 2 diabetes mellitus. Curr. Med. Sci..

[8] Chauhan A., Patel S.S. (2024). Thyroid Hormone and Diabetes Mellitus Interplay: Making Management of Comorbid Disorders Complicated. Horm. Metab. Res..

[9] Onitilo A.A., Engel J.M., Glurich I., Stankowski R.V., Williams G.M., Doi S.A. (2012). Diabetes and cancer II: Role of diabetes medications and influence of shared risk factors. Cancer Causes Control..

[10] Yeo Y., Ma S.H., Hwang Y., Horn-Ross P.L., Hsing A., Lee K.E., Park Y.J., Park D.J., Yoo K.Y., Park S.K. (2014). Diabetes mellitus and risk of thyroid cancer: A meta-analysis. PLoS ONE.

[11] Hussein S.M.M., AbdElmageed R.M. (2021). The relationship between type 2 diabetes mellitus and related thyroid diseases. Cureus.

[12] Soriguer F., Gutiérrez-Repiso C., Rubio-Martin E., Linares F., Cardona I., López-Ojeda J., Pacheco M., González-Romero S., Garriga M.J., Velasco I. (2011). Iodine intakes of 100-300 μg/d do not modify thyroid function and have modest anti-inflammatory effects. Br. J. Nutr..

[13] Macvanin M.T., Gluvic Z., Zafirovic S., Gao X., Essack M., Isenovic E.R. (2023). The protective role of nutritional antioxidants against oxidative stress in thyroid disorders. Front. Endocrinol..

[14] Moura Neto A., Parisi M.C., Tambascia M.A., Pavin E.J., Alegre S.M., Zantut-Wittmann D.E. (2014). Relationship of thyroid hormone levels and cardiovascular events in patients with type 2 diabetes. Endocrine.

[15] Xing M. (2012). Oxidative stress: A new risk factor for thyroid cancer. Endocr.-Relat. Cancer.

[16] Zhang C., Gao X., Han Y., Teng W., Shan Z. (2021). Correlation Between Thyroid Nodules and Metabolic Syndrome: A Systematic Review and Meta-Analysis. Front. Endocrinol..

[17] Hadgu R., Worede A., Ambachew S. (2024). Prevalence of thyroid dysfunction and associated factors among adult type 2 diabetes mellitus patients, 2000–2022: A systematic review and meta-analysis. Syst. Rev..

[18] Tiwari A., Balasundaram P. (2024). Public Health Considerations Regarding Obesity. [Updated 5 June 2023]. StatPearls [Internet].

[19] Li L., Liu S., Yu J. (2020). Autoimmune thyroid disease and type 1 diabetes mellitus: Same pathogenesis; new perspective?. Ther. Adv. Endocrinol. Metab..

[20] Nederstigt C., Corssmit E.P., de Koning E.J., Dekkers O.M. (2016). Incidence and prevalence of thyroid dysfunction in type 1 diabetes. J. Diabetes Complicat..

[21] Orzan A., Novac C., Mihu M., Tirgoviste C.I., Balgradean M. (2016). Type 1 Diabetes and Thyroid Autoimmunity in Children. Maedica.

[22] Giovannucci E.L., Harlan D.M., Archer M.C., Bergenstal R.M., Gapstur S.M., Habel L.A., Pollak M., Regensteiner J.G., Yee D. (2010). Diabetes and cancer: A consensus report. Diabetes Care.

[23] Zhu B., Qu S. (2022). The Relationship Between Diabetes Mellitus and Cancers and Its Underlying Mechanisms. Front. Endocrinol..

[24] Kadiyala R., Peter R., Okosieme O.E. (2010). Thyroid dysfunction in patients with diabetes: Clinical implications and screening strategies. Int. J. Clin. Pract..

[25] Baltzis D., Eleftheriadou I., Veves A. (2014). Pathogenesis and Treatment of Impaired Wound Healing in Diabetes Mellitus: New Insights. Adv. Ther..

[26] Palma C.C.S.S.V., Pavesi M., Nogueira V.G., Clemente E.L.S., Vasconcellos M.d.F.B.M.P., Pereira L.C., Pacheco F.F., Braga T.G., Bello L.d.F., Soares J.O. (2013). Prevalence of thyroid dysfunction in patients with diabetes mellitus. Diabetol. Metab. Syndr..

[27] Vondra K., Vrbikova J., Dvorakova K. (2005). Thyroid gland diseases in adult patients with diabetes mellitus. Minerva Endocrinol..

[28] Biondi B., Kahaly G.J., Robertson R.P. (2019). Thyroid Dysfunction and Diabetes Mellitus: Two Closely Associated Disorders. Endocr. Rev..

[29] Gierach M., Gierach J., Junik R. (2014). Insulin resistance and thyroid disorders. Endokrynol. Pol..

[30] Spira D., Buchmann N., Dörr M., Markus M.R.P., Nauck M., Schipf S., Spranger J., Demuth I., Steinhagen-Thiessen E., Völzke H. (2022). Association of thyroid function with insulin resistance: Data from two population-based studies. Eur. Thyroid J..

[31] Rong F., Dai H., Wu Y., Li J., Liu G., Chen H., Zhang X. (2021). Association between thyroid dysfunction and type 2 diabetes: A meta-analysis of prospective observational studies. BMC Med..

[32] Yin D.T., He H., Yu K., Xie J., Lei M., Ma R., Li H., Wang Y., Liu Z. (2018). The association between thyroid cancer and insulin resistance, metabolic syndrome and its components: A systematic review and meta-analysis. Int. J. Surg..

[33] Zhao J., Zhang Q., Yang Y., Yao J., Liao L., Dong J. (2021). High prevalence of thyroid carcinoma in patients with insulin resistance: A meta-analysis of case-control studies. Aging.

[34] Liu W., Sun Y., Zhang Y., Yin D. (2025). The causal relationships between inflammatory cytokines, blood metabolites, and thyroid cancer: A two-step Mendelian randomization analysis. Discov. Oncol..

[35] Le T.N., Bright R., Truong V.K., Li J., Juneja R., Vasilev K. (2025). Key biomarkers in type 2 diabetes patients: A systematic review. Diabetes Obes. Metab..

[36] Crnčić T.B., Tomaš M.I., Girotto N., Ivanković S.G. (2020). Risk Factors for Thyroid Cancer: What Do We Know So Far?. Acta Clin. Croat..

[37] Matei M., Vlad M.M., Golu I., Dumitru C.Ș., De Scisciolo G., Matei S.-C. (2023). Can Routine Laboratory Tests Be Suggestive in Determining Suspicions of Malignancy in the Case of Thyroid Nodules?. Medicina.

[38] Zhao J., Tian Y., Jia Z., Yao J., Liao L., Dong J. (2022). Abnormal Glucose Metabolism Parameters and the Aggressiveness of Differentiated Thyroid Carcinoma: A Hospital-Based Cross-Section Study in China. Front. Endocrinol..

[39] Wu R., Zhang J., Zou G., Li S., Wang J., Li X., Xu J. (2024). Diabetes Mellitus and Thyroid Cancers: Risky Correlation, Underlying Mechanisms and Clinical Prevention. Diabetes Metab. Syndr. Obes..

[40] Bhatti J.S., Sehrawat A., Mishra J., Sidhu I.S., Navik U., Khullar N., Kumar S., Bhatti G.K., Reddy P.H. (2022). Oxidative stress in the pathophysiology of type 2 diabetes and related complications: Current therapeutics strategies and future perspectives. Free Radic. Biol. Med..

[41] Kochman J., Jakubczyk K., Bargiel P., Janda-Milczarek K. (2021). The Influence of Oxidative Stress on Thyroid Diseases. Antioxidants.

[42] Elumalai S., Karunakaran U., Moon J.S., Won K.C. (2021). NADPH Oxidase (NOX) Targeting in Diabetes: A Special Emphasis on Pancreatic β-Cell Dysfunction. Cells.

[43] Dang H., Sheng J., Tang P., Peng X., Zhang R., Zhao X., Hu J., Xu T. (2023). The role and mechanism of NADPH oxidase in the development and progression of thyroid carcinoma. Am. J. Cancer Res..

[44] Kościuszko M., Buczyńska A., Krętowski A.J., Popławska-Kita A. (2023). Could Oxidative Stress Play a Role in the Development and Clinical Management of Differentiated Thyroid Cancer?. Cancers.

[45] Wang D., Feng J.F., Zeng P., Yang Y.H., Luo J., Yang Y.W. (2011). Total oxidant/antioxidant status in sera of patients with thyroid cancers. Endocr.-Relat. Cancer.

[46] Gao J., Liu J. (2024). Correlation of serum thyrotropin and thyroid hormone levels with diabetic kidney disease: A cross-sectional study. BMC Endocr. Disord..

[47] Tang Y., Yan T., Wang G., Chen Y., Zhu Y., Jiang Z., Yang M., Li C., Li Z., Yu P. (2017). Correlation between Insulin Resistance and Thyroid Nodule in Type 2 Diabetes Mellitus. Int. J. Endocrinol..

[48] Gu Y., Li H., Bao X., Zhang Q., Liu L., Meng G., Wu H., Du H., Shi H., Xia Y. (2017). The Relationship Between Thyroid Function and the Prevalence of Type 2 Diabetes Mellitus in Euthyroid Subjects. J. Clin. Endocrinol. Metab..

[49] Shang J., Zheng Y., Zhang M., Li M., Qiang W., Sui J., Guo H., Shi B., He M. (2024). Lower Free Thyroxine Levels Are Associated with Diabetic Kidney Disease in Males with Type 2 Diabetes Mellitus: An Observational Cross-Sectional Study. Biomedicines.

[50] Zhao X., Sun J., Xin S., Zhang X. (2023). Predictive Effects of FT3/FT4 on Diabetic Kidney Disease: An Exploratory Study on Hospitalized Euthyroid Patients with T2DM in China. Biomedicines.

[51] Nanu M., Delia C.E., Toma G.M., Ardeleanu I., Nanu I., Stemate M., Nuta D., Gheorghiu M.L. (2024). Iodine Status in Romania After 20 Years of Mandatory Salt Iodization: Discordant Results in Schoolchildren and Neonates. Acta Endocrinol..

[52] Karakaya R.E., Saka M., Ozdemir D. (2020). Determining the relationship between dietary iodine intake, urinary iodine excretion and thyroid functions in people with type 2 diabetes mellitus. Arch Endocrinol. Metab..

[53] Chen D.W., Lang B.H.H., McLeod D.S.A., Newbold K., Haymart M.R. (2023). Thyroid cancer. Lancet.

[54] Borlea A., Moisa-Luca L., Popescu A., Bende F., Stoian D. (2024). Combining CEUS and ultrasound parameters in thyroid nodule and cancer diagnosis: A TIRADS-based evaluation. Front. Endocrinol..

[55] Borlea A., Borcan F., Sporea I., Dehelean C.A., Negrea R., Cotoi L., Stoian D. (2020). TI-RADS Diagnostic Performance: Which Algorithm Is Superior and How Elastography and 4D Vascularity Improve the Malignancy Risk Assessment. Diagnostics.

[56] Streinu D.R., Neagoe O.C., Borlea A., Icma I., Derban M., Stoian D. (2024). Enhancing diagnostic precision in thyroid nodule assessment: Evaluating the efficacy of a novel cell preservation technique in fine-needle aspiration cytology. Front. Endocrinol..

[57] Dumitru C.S., Ceausu A.R., Comsa S., Raica M. (2022). Loss of E-Cadherin Expression Correlates With Ki-67 in Head and Neck Squamous Cell Carcinoma. In Vivo.

[58] Dumitru C.S., Raica M. (2023). Vascular Endothelial Growth Factor Family and Head and Neck Squamous Cell Carcinoma. Anticancer Res..

[59] Cosoroabă R.M., Gaje N.P., Ceauşu A.R., Dumitru C.Ş., Todor L., Popovici R.A., Porumb A., Domocoş D., Miron M.I. (2022). The mast cell reaction in premalignant and malignant lesions of the head and neck. Rom. J. Morphol. Embryol..

[60] Dumitru C.S., Raica M. (2024). A Splice Form of VEGF, a Potential Anti-Angiogenetic Form of Head and Neck Squamous Cell Cancer Inhibition. Int. J. Mol. Sci..

[61] Dumitru C.S., Ceausu A.R., Gaje N.P., Suciu C.S., Raica M. (2022). Proliferating Lymphatic Endothelial Cells as a Prognostic Marker in Head and Neck Squamous Cell Carcinoma. Int. J. Mol. Sci..

[62] Mehran L., Delbari N., Amouzegar A., Hasheminia M., Tohidi M., Azizi F. (2022). Reduced Sensitivity to Thyroid Hormone Is Associated with Diabetes and Hypertension. J. Clin. Endocrinol. Metab..

[63] Schiller A., Gadalean F., Schiller O., Timar R., Bob F., Munteanu M., Stoian D., Mihaescu A., Timar B., Seguro A.C. (2015). Vitamin D deficiency—prognostic marker or mortality risk factor in end stage renal disease patients with diabetes mellitus treated with hemodialysis—A prospective multicenter study. PLoS ONE.

[64] Ginoudis A., Ioannidou S., Tsakiroglou G., Kazeli K., Vagdatli E., Lymperaki E. (2024). Correlation of Albumin, Red Cell Distribution Width and Other Biochemical and Hematological Parameters with Glycated Hemoglobin in Diabetic, Prediabetic and Non-Diabetic Patients. Int. J. Mol. Sci..

[65] National Guideline Centre (UK) (2019). Thyroid Function tests: Thyroid Disease: Assessment and Management.

[66] Bosma M., Du Puy R.S., Ballieux B.E.P.B. (2022). Screening for thyroid dysfunction with free T4 instead of thyroid stimulating hormone (TSH) improves efficiency in older adults in primary care. Age Ageing.

